# Complete Genome Sequence of Lactobacillus plantarum Strain JDARSH, Isolated from Sheep Milk

**DOI:** 10.1128/MRA.01199-19

**Published:** 2020-01-09

**Authors:** Abhinandan Patil, Anamika Dubey, Muneer Ahmad Malla, John Disouza, Shivaji Pawar, Abdulaziz A. Alqarawi, Abeer Hashem, Elsayed Fathi Abd_Allah, Ashwani Kumar

**Affiliations:** a Centre for Interdisciplinary Research, D. Y. Patil University, Kolhapur, Maharashtra, India; b Centre for Innovative and Applied Research, Anekant Education Society, Tuljaram Chaturchand College, Baramati, Maharashtra, India; c Metagenomics and Secretomics Research Lab, Department of Botany, Dr. Harisingh Gour University (a Central University), Sagar, Madhya Pradesh, India; d Department of Zoology, Dr. Harisingh Gour University (a Central University), Sagar, Madhya Pradesh, India; e Plant Production Department, College of Food and Agriculture Science, King Saud University, Riyadh, Saudi Arabia; f Botany and Microbiology Department, College of Science, King Saud University, Riyadh, Saudi Arabia; g Mycology and Plant Disease Survey Department, Plant Pathology Research Institute, Agriculture Research Center, Giza, Egypt; Loyola University Chicago

## Abstract

Lactobacillus plantarum strain JDARSH, a potential probiotic with a wide range of functions, was isolated from sheep milk. Here, we report the whole-genome sequence of this bacterium. The draft genome yielded a 3.20-Mb genome and 2,980 protein-coding sequences.

## ANNOUNCEMENT

Lactic acid bacteria (LAB) are widely used in various preparations, such as food and food products, and for other raw materials, including vegetables, meat, and plant products. A number of recently conducted studies have led to the conviction that some strains of LAB, in particular, strains from the genus Lactobacillus, may promote health in both humans and animals (1–3); among these, the species Lactobacillus plantarum is the most flexible and versatile. It is a Gram-positive, facultatively anaerobic, rod-shaped, acid-tolerant, and non-spore-forming probiotic bacterium (4–6). The bacterium has wide application in the medical field (7, 8). In this study, we have sequenced the whole genome of an L. plantarum strain isolated from sheep milk.

A total of 180 sheep milk samples (Indian breed) were collected from local places in the Kolhapur, Sangli, and Admapur areas of Maharashtra, India. For bacterial enumeration, milk samples (1 ml) were kept at −80°C in 15% glycerol before use. Isolation and purification were performed on de Man-Rogosa-Sharpe (MRS) broth and agar medium (9). The samples were inoculated on MRS agar medium and incubated for a period of 48 h under microaerophilic conditions. After incubation, the individual colonies formed were transferred into sterile MRS broth medium. The culture is deposited in the public domain as Lactobacillus plantarum strain JDARSH at the National Centre for Microbial Resources (NCMR), Pune, India. Total DNA was extracted using a PureLink genomic DNA extraction kit (Life Technologies), following the manufacturer’s instructions. The sequencing libraries were prepared using a Nextera XT library kit, and whole-genome sequencing of *L. plantarum* strain JRARSH was performed on the Illumina NextSeq 500 sequencing platform (150-bp paired-end reads) with a shotgun strategy (9). A total of 1,203,568 paired-end reads of 150 bp in size were generated. The Illumina-generated sequence reads were quality filtered by FastQC v.0.10.1 (http://www.bioinformatics.babraham.ac.uk/projects/fastqc/), and low-quality reads were removed before assembly. The quality-filtered reads were assembled *de novo* using SPAdes v.3.9 (10, 11), producing 31 contigs. The gene prediction was performed using the Rapid Annotations using Subsystems Technology (RAST) server v.2.0 (rast.nmpdr.org/), with default parameters (12–14). Predicted genes were annotated using the Prokaryotic Genome Annotation Pipeline (PGAP) v.4.9 (15) and BLAST (http://blast.ncbi.nlm.nih.gov/Blast.cgi). Default parameters were used for all software, unless otherwise specified. The final assembly yielded a 3.20-Mb genome with a mean G+C content of 44.5%, an *N*_50_ value of 174,093 bp, an *L*_50_ of 6, and a total of 3,128 genes, of which 2,980 are protein-coding genes, 59 are tRNA genes, 5 are rRNA genes (35S rRNAs, 116S rRNA, and 123S rRNA), and 4 are noncoding RNA (ncRNA) genes. The genome also contained 80 pseudogenes. Within the total of 3,128 genes, biological functions were defined for 2,135 (68.25%). The predicted genes that were identified are associated with cellular metabolism (*n* = 38), protein processing (*n* = 323), energy (*n* = 276), stress response defense and virulence (*n* = 182), DNA processing (*n* = 181), cellular response (*n* = 163), RNA processing (91), membrane transport (*n* = 33), the cellular envelope (*n* = 28), and regulation and cell signaling (*n* = 18). Moreover, the genes were each assigned a putative function. Putative functions included cell adhesions, acid tolerance, bile toxicity, molybdenum cofactor biosynthesis, folate and pterine biosynthesis, aromatic compound degradation, exopolysaccharide production, riboflavin and bacteriocin production, thiamine and methionine biosynthesis, phosphate metabolism, sulfur metabolism, dormancy and sporulation, and denitrification. Similar pathways were also found in some other strains of *L. plantarum* (16–18). This Lactobacillus plantarum bacterial strain also proves to be an attractive candidate for the metabolic engineering of lignocellulosic biomass to biofuels, owing to its characteristic natural abilities to metabolize the hexose sugars and to tolerate high ethanol and acid concentrations (19, 20).

### Data availability.

The associated BioSample, SRA, and BioProject accession numbers for the sequence reported here are SAMN13106942, SRP226774, and PRJNA579228, respectively.
